# Sex differences in a Brazilian sample of patients with inflammatory bowel disease

**DOI:** 10.1590/1806-9282.20240963

**Published:** 2024-12-02

**Authors:** Khadija Assis Pascholatto, Laura Ribeiro Santos, Thelma Larocca Skare, Odery Ramos, Renato Nisihara

**Affiliations:** 1 Mackenzie Evangelical School of Medicine of Paraná – Curitiba (PR), Brazil.; 2 Universidade Federal do Paraná, Department of Clinical Medicine – Curitiba (PR), Brazil.

**Keywords:** Inflammatory bowel disease, Sex, Clinical presentation

## Abstract

**BACKGROUND:**

Inflammatory bowel diseases such as Crohn’s disease and ulcerative colitis are influenced by environmental and immunological factors and may differ according to the patient’s sex.

**OBJECTIVE:**

The objective was to study the differences in the clinical profile of a Brazilian sample of inflammatory bowel disease patients according to sex.

**METHODS:**

Retrospective study with chart review of 158 inflammatory bowel disease patients (43 with Crohn’s disease and 115 with ulcerative colitis) from a single university hospital in southern Brazil.

**RESULTS:**

The Crohn’s disease sample showed a female/male ratio of 2.1, and the sample of ulcerative colitis showed a ratio of 1.5. The only significant difference found in the clinical profile was an increased constipation rate in female patients with ulcerative colitis. No other differences in epidemiological, symptom profile, or treatment could be detected.

**CONCLUSIONS:**

More females with inflammatory bowel diseases sought healthcare facilities compared to males. The only notable difference was a higher incidence of constipation symptoms among females; all other aspects were similar between the sexes.

## INTRODUCTION

Inflammatory bowel diseases (IBDs) are chronic disorders with autoimmune features that include two major disorders: ulcerative colitis (UC) and Crohn’s disease (CD). While UC affects only the colon, CD involves the whole gastrointestinal tract^1^.

The IBD prevalence has been growing worldwide, mainly in newly industrialized countries. In Taiwan, the annual percentage change increased by 4.0% (95%CI 1.0–7.1) for CD and 4.8% (95%CI 1.8–8.0) for UC. In Brazil, it increased by 11.1% (95%CI 4.8–17.8) for CD and by 14.9 % (95%CI 10.4–19.6) for UC^2^.

Genetic, environmental, and lifestyle factors may be linked to its appearance^3,4,5^. Immune response has also been implicated in their etiology with participation of both adaptive and innate systems^3,6,7^.

Immune response has been subject to variations according to individual’s sex. The X chromosome and sexual hormones such as estrogens, prolactin, and testosterone are active players in this context^8^. Incidence rates of IBD according to sex points toward 1:1.5 in CD and 1:1.2 UC^1,9,10^. Shah et al.^11^ reported that female patients under 14 years of age had a lower risk of CD but a higher risk than males thereafter; they also described that UC had the same prevalence until the age of 45 years old, with male predominance after this age. According to Wagtmans et al.^9^, female sex has been described as a risk factor for extraintestinal manifestations (EIMs) in CD. Others observed that males were more likely to present with upper gastrointestinal involvement and to have small bowel surgery^12,13^. Khrom et al.^14^ found that males were more likely to have perianal disease, colonic-only disease location in CD, and also more extensive disease in UC. Moreover, females with IBD have been shown to be more anxious^15^ and to have more healthcare consumption than males^16^.

Studies of the IBD clinical pattern according to sex are rare, mainly in Brazilian samples.

Herein, we studied a sample of Brazilian patients with IBD, aiming to know the possible differences in the symptom profile at disease diagnosis according to patient’s sex.

## METHODS

### Ethical issues

This is a retrospective study approved by the institutional committee of ethics in research CAAE-52867521.3.0000.0103, under protocol number 5.021.415. Medical charts from a gastroenterology service, in a single tertiary hospital that cares for patients from the public health system in Southern Brazil, from January 2018 to September 2021 were reviewed.

### Participants

To be included, patients should have been diagnosed and classified as IBD according to ECCO (European Crohn’s and Colitis Organization)/ESGAR (European Society of Gastrointestinal and Abdominal Radiology) Guidelines for Diagnostic Assessment in IBD^13,14^.

### Data collection at diagnosis

Epidemiological data: sex, age, age at diagnosis.

Clinical data: IBD type, disease location according to the endoscopic exam, symptoms (diarrhea, weight loss, anorexia, abdominal pain, rectal bleeding, mucus in the stool, constipation, melena, hematochezia, and tenesmus), used treatment, and presence of EIMs and neoplasia.

Disease activity: Crohn’s disease activity index (CDAI) and Harvey-Bradshaw index^17,18^ for patients with CD and Mayo score for patients with UC^17,18^.

### Statistical analysis

For statistical analysis, data on males and females were compared using the GraphPad Prism version 9.5.1 software for Windows, GraphPad Software, San Diego, California, USA (www.graphpad.com). Chi-squared and Fisher tests were used for nominal variables and unpaired t-test and Mann-Whitney for numerical variables according to data distribution. Data distribution was judged by the Shapiro-Wilk test. The adopted significance was 5%.

## RESULTS

A total of 158 IBD patients were studied, with 48 being diagnosed with CD and 115 with UC. Table 1 shows the clinical profile at diagnosis of the studied patients. The CD sample showed a female/male ratio of 2.1, and the sample of UC showed a female/male ratio of 1.5.

**Table 1:** Description of patients with inflammatory bowel disease patients studied (n=158).

	Ulcerative colitis n=115	Crohn’s disease n=43
Female/male (ratio)	70/45 (1.5:1)	29/14 (2.1:1)
Mean age (SD)—years	48.5 (16.0)	46.7 (16.2)
Mean age at diagnosis (SD)—years	39.5 (15.3)	37.0 (16.1)
Median disease duration (IQR)	8 (5–13)	10 (4–14)
Tobacco exposure—n (%)	20 (17.3%)	4 (9.3)
Clinical presentation—n (%)		
Diarrhea	64 (55.6)	22 (51.1)
Constipation	11 (9.5)	5 (11.6)
Weight loss	14 (12.1)	10 (23.2)
Abdominal pain	50 (43.4)	14 (32.5)
Rectal bleeding	9 (7.8)	4 (9.3)
Stool mucus	29 (25.2)	4 (9.3)
Melena	0	7 (16.2)
Tenesmus	11 (9.5)	0
Extraintestinal manifestations n (%)		
Fistula	3 (2.6)	20 (46.5)
Hemorrhoids	22 (19.1)	9 (20.9)
Anal abscess	1 (0.8)	2 (4.6)
Joint manifestations	4 (3.4)	2 (4.6)
Treatment n (%)		
Mesalamine	98 (85.2)	8 (18.6)
Azathioprine	22 (19.1)	24 (55.8)
Anti-TNF alpha	10 (8.6)	13 (30.2)
Vedolizumab	4 (3.4)	0
Prednisone	5 (4.3)	1 (2.3)
Previous surgery	10 (8.6)	26 (60.4)
Gastrointestinal neoplasm	2 (1.7)	3 (6.9)

SD: standard deviation;. IQR: interquartile range; TNF: tumor necrosis factor.

The comparison of epidemiological, clinical, and treatment factors in males and females for each IBD is given in Table 2. This table shows that from the clinical point of view, only constipation was more commonly found in females with UC. A tendency toward more rectal bleeding in males with CD and more fistulas in males with UC was also seen, but the number of occurrences was too low to allow any conclusion.

**Table 2:** Comparison of epidemiological and clinical data in males and females with inflammatory bowel diseases.

	Ulcerative colitis			Crohn’s disease		
	Males n=45	Females n=70	p	Males n=14	Females n=29	p
Mean age (SD)—years	48.0 (17.5)	48.8 (15.0)	0.77	44.5 (15.3)	47.7 (16.7)	0.54
Median disease duration (IQR)—years	9.0 (4.0–15.0)	7.0 (5.0–11.0)	0.74	9 (3.7–14.2)	10 (5.0–14.5)	0.68
Mean age at disease onset (SD)—years	39.3 (16.4)	39.7 (14.7)	0.90	33.5 (16.2)	38.7 (16.0)	0.32
Clinical aspects—n (%)						
Diarrhea	28 (62.2)	36 (51.4)	0.33	9 (64.2)	13 (44.8)	0.33
Constipation	1 (2.2)	10 (14.2)	0.04	2 (14.2)	3 (10.3)	0.99
Abdominal pain	16 (35.5)	34 (48.5)	0.16	4 (28.5)	10 (34.4)	0.99
Weight loss	6 (13.3)	8 (11.4)	0.76	5 (35.7)	5 (17.2)	0.25
Rectal bleeding	4 (8.8)	5 (7.1)	0.73	7 (50)	2 (6.8)	0.07
Extraintestinal manifestations						
Fistulas	3 (6.6)	0	0.059	9 (64.2)	11 (37.9)	0.19
Hemorrhoids	8 (17.7)	14 (20)	0.76	2 (14.2)	7 (24.1)	0.69
Anal abscess	1 (2.2)	0	0.39	2 (14.2)	0	0.10
Treatment requirement—n (%)						
Mesalamine	41 (91.1)	57 (81.4)	0.18	2 (14.2)	6 (55.1)	0.99
Azathioprine	5 (11.1)	17 (24.2)	0.09	10 (71.4)	14 (48.2)	0.19
Anti-TNF alpha	10 (22.2)	5 (7.1)	0.50	4 (28.5)	9 (31.0)	0.99
Glucocorticoid	2 (4.4)	3 (4.2)	0.99	0	1 (3.4)	2

SD: standard deviation. IQR: interquartile range; TNF: tumor necrosis factor.

When comparing sexes, no differences were found in disease activity in CD measured through CDAI (p=0.66) nor in UC measured by the Mayo score (p=0.86).

## DISCUSSION

Studying the sex influence in IBD phenotype is important as this disease suffers from the influence of environmental and immunological factors^19^. Environmental trigger factors such as tobacco use may diverge according to patients’ sex, although the rates may vary worldwide. The X chromosome is known to carry genes with an important role in the immune system such as genes for TLR-7, TLR-9, the transcriptional factor forkhead box P3, the chemokine receptor CXCR3, CD40L, and interleukin 9 (IL-9) that influence cytokine production like TNF-alpha and interferon-alpha^8^. A greater IBD incidence rate in patients with Turner’s syndrome (XO genotype) suggests the influence of the X chromosome on this setting^16^. Also, sexual hormones such as testosterone have immunosuppressive effects, while estrogens seem to be responsible for higher immune responses in women^8^. The use of contraceptive pills is linked to the risk and with the progression of CD; estrogen is known to have a pro-inflammatory effect^11,20^.

The results of this analysis show that females with IBD seek more consultation mainly those with CD. Targownik et al.^16^ also found a higher health care consumption by females in a large cohort of Canadians with IBD. From the clinical point of view, males and females with similar disease duration had a comparable symptom profile except for the presence of more constipation in females with UC. Previous works have found a higher prevalence of EIMs in females. Currently, this aspect could not be proven. However, the number of extraintestinal occurrences was quite small and could have precluded a correct interpretation.

In this sample, males and females have had IBD diagnoses at a similar age. A study conducted in a Chinese population found that males are diagnosed at a younger age than females^20^, but another study, from the Netherlands, found the opposite, indicating that females experienced the disease earlier^9^. Racial differences in disease expression and economic aspects that influence access to health care may explain this variation.

Presently, the used treatment was similar in both sexes, including the need for surgery. Trindade et al.^21^ in a study of 200 individuals observed a higher rate of surgical procedures in males with both IBDs, while Liu et al.^20^ found that males required more surgery than females with CD. Nevertheless, Wagtmans et al.^9^ in their study on CD found no differences according to sex in surgery requirement as in the present study.

The present work has limitations due to its retrospective design and the small number of included IBD patients. However, it shows the real-life scenario in a Brazilian public health center.

In conclusion, in this sample, more females with IBD looked for health care facilities than males. Except for more constipation symptoms in females, all other aspects were ­similar in both sexes.
